# Possible connections of the opposite trends in Arctic and Antarctic sea-ice cover

**DOI:** 10.1038/srep45804

**Published:** 2017-04-05

**Authors:** Lejiang Yu, Shiyuan Zhong, Julie A. Winkler, Mingyu Zhou, Donald H. Lenschow, Bingrui Li, Xianqiao Wang, Qinghua Yang

**Affiliations:** 1 Polar Research Institute of China, Shanghai, China; 2 State Oceanic Administration Key Laboratory for Polar Science, Shanghai, China; 3 Department of Geography, Environment and Spatial Sciences, Michigan State University, East Lansing, MI, USA; 4 Key Laboratory of Research on Marine Hazards Forecasting, National Marine Environment Forecast Center, Beijing, China; 5 National Center for Atmospheric Research, Boulder, CO, USA

## Abstract

Sea ice is an important component of the global climate system and a key indicator of climate change. A decreasing trend in Arctic sea-ice concentration is evident in recent years, whereas Antarctic sea-ice concentration exhibits a generally increasing trend. Various studies have investigated the underlying causes of the observed trends for each region, but possible linkages between the regional trends have not been studied. Here, we hypothesize that the opposite trends in Arctic and Antarctic sea-ice concentration may be linked, at least partially, through interdecadal variability of the Pacific Decadal Oscillation (PDO) and the Atlantic Multidecadal Oscillation (AMO). Although evaluation of this hypothesis is constrained by the limitations of the sea-ice cover record, preliminary statistical analyses of one short-term and two long-term time series of observed and reanalysis sea-ice concentrations data suggest the possibility of the hypothesized linkages. For all three data sets, the leading mode of variability of global sea-ice concentration is positively correlated with the AMO and negatively correlated with the PDO. Two wave trains related to the PDO and the AMO appear to produce anomalous surface-air temperature and low-level wind fields in the two polar regions that contribute to the opposite changes in sea-ice concentration.

Sea ice, an important component of the polar climate system, is a prominent indicator of global climate change^1^,^2^,^3^,^4^,^5^. The recent decline of Arctic sea ice has received considerable attention^3^,^6^, as it has a significant impact on the Arctic climate and ecosystem^7^, as well as on mid-latitude weather and climate^8^. Various studies have investigated possible causes for the Arctic sea-ice decline and a number of physical processes are believed to be responsible^2^,^8^,^9^, including atmospheric thermodynamic^9^ and dynamic forcing^10^, oceanic heat transport^11^, and the Arctic amplification of climate warming^12^ associated with ice-albedo feedback^13^ and water-vapor and cloud radiative feedback^14^. Studies have also attributed the Arctic sea-ice decline to several global atmosphere-ocean circulation anomalies including the Arctic Dipole (AD)^15^, Atlantic Multidecadal Oscillation (AMO)^16^, the Arctic Oscillation (AO)^17^, the North Atlantic Oscillation (NAO)^18^,and the Pacific Decadal Oscillation (PDO)^19^,^20^.

In contrast to the declining trends in Arctic sea ice, Antarctic sea-ice cover has exhibited an overall increasing trend in the past few decades^3^,^4^, except for the Bellingshausen and Amundsen Seas region where there has been a significant decrease^21^. Although it has been suggested that stratospheric ozone depletion that strengthened cyclonic circulation over Antarctica contributed to the Antarctic sea ice expansion^22^, several studies have linked the increasing Antarctic sea ice cover to internally generated variability forced from the tropical Pacific that deepened the Amundsen Sea Low^23^,^24^,^25^,^26^,^27^,^28^,^29^. Recent research has shown that AMO also is an important factor leading to the complex distribution of Antarctic sea ice^30^,^31^,^32^,^33^, and that the Interdecadal Pacific Oscillation (IPO) has contributed to recent sea-ice expansion^29^. A recent study found that the trends in Antarctic sea-ice cover are partly forced by sea-surface temperature anomalies in the north and tropical Atlantic, through changes in the global thermohaline circulation and sea-level change^31^,^33^. Another potential mechanism for the Antarctic sea-ice expansion is that increased mass loss of the Antarctic ice sheet due to increasing ocean warming and subsurface ice-shelf melting causes the uppermost ocean layer to become fresher and thus less dense than the underlying warmer water and this decreases upward ocean heat transport. The upper ocean layer can then cool more rapidly in the autumn and early winter, thus leading to the sea-ice expansion^34^.

The associations found in previous studies, whether for the Arctic or Antarctic, between trends in sea-ice cover and low-frequency global climate oscillations such as the AMO, PDO and IPO suggest a possible connection between the opposing trends in sea-ice cover in the two polar regions. Most previous studies, however, have examined separately either the decreasing Arctic or the increasing Antarctic sea ice cover, rather than a more comprehensive examination of global sea-ice cover that concurrently considers trends in the Arctic and Antarctic. We hypothesize that the opposing trends in sea-ice cover in the two polar regions are, in part, connected via low-frequency global modes of climate variability, and provide an initial evaluation of this hypothesis. We focus on two indices of climate variability, the AMO and PDO, which capture atmosphere-ocean anomalies in the Atlantic and Pacific, respectively. Statistical analysis is used to assess the degree of association between time series of the AMO and PDO indices and sea-ice concentration. We employ both short-term remotely-sensed observations of sea-ice concentration and century-long estimates obtained from observational datasets and atmospheric reanalyses.

We first focus on the concurrent temporal trends in high-resolution, remotely-sensed Arctic and Antarctic sea-ice concentrations obtained from the National Snow and Ice Data Center (NSIDC) for the period 1979 through 2013. Empirical orthogonal function (EOF) analyses are performed to jointly identify for the two polar regions (50°–90°N and 50°–90°S) the prevailing spatial patterns of the variability of seasonal mean sea-ice concentration for MAM (March-May), JJA (June-August), SON (September-November) and DJF (December-February). The sea-ice concentration time series were not detrended or filtered prior to the EOF analysis because of the short length of the NSIDC observational record, especially in comparison to the low-frequency PDO and AMO climate anomalies. The percentages of the total variance explained by the first EOF mode (EOF1) range from 16% for DJF to 22% for MAM. The EOF1 spatial patterns (Fig. 1a) show negative sea-ice anomalies in the Arctic Ocean with the exception of a small area over the Bering Sea where positive anomalies occur in MAM and DJF. Negative anomalies appear over the Barents Sea, the Greenland Sea and the Sea of Okhotsk in DJF and MAM, which expand to cover larger areas of the Arctic Ocean in JJA and SON. Both positive and negative anomalies occur over the Antarctic Ocean and, although there is considerable seasonal variation, more areas are covered by positive than negative anomalies. In DJF and MAM, negative Antarctic sea-ice anomalies occur in the Bellingshausen, Amundsen and eastern Ross Seas, while positive anomalies are seen in the other coastal seas. In JJA and SON, negative anomalies occur in the Bellingshausen and Weddell Seas and the southern Indian Ocean near 90°E, and positive anomalies occur elsewhere.

The time coefficients, or principal components (PC), of EOF1 show an overall increasing trend (significant at the 99% confidence level) for all four seasons (Fig. 1b), with considerable temporal variability on both the interannual and interdecadal time scales. The spatial patterns of the portion of the total (i.e., observed) trends that is not explained by EOF1 (i.e., the residual trends) can be estimated by subtracting the PC1 trends from the total trends. A comparison of the magnitude of the total trends (Fig. 2) with those of the residual trends (Fig. 3) indicates that more than two-thirds of the total trends can be explained by the PC1 trends. Moreover, the residual trends (Fig. 3) show different spatial patterns from the total trends. For example, over the Arctic, large negative residual trends in MAM and DJF occur in the Davis Strait and Labrador Sea. Oceanic processes related to the West Greenland Current may determine the decadal variation of sea ice in the Labrador Sea^35^. Over the Antarctic, negative residual trends for MAM and DJF occur mainly in the Weddell Sea, which is different from the negative total trends in the Bellingshausen, Amundsen and eastern Ross Seas. Although JJA and SON have similar spatial patterns for the total trend, their residual trend patterns differ considerably particularly over the Ross and Weddell Seas. In sum, the similarity between the spatial patterns of the EOF1 anomalies and those of the observed trends obtained from the NSIDC remotely-sensed data, along with the small residual trends and their differing patterns from the total trends, support the use of PC1 as a composite measure of the temporal trend in global sea-ice concentrations.

The PC1 time series is compared to the AMO and PDO time series, both of which display significant (99.9% confidence interval) overall trends, positive for AMO and negative for PDO (Table 1), although also with large interannual and interdecadal fluctuations (Fig. 1b). All three trend lines (PC1, AMO, PDO) cross the horizontal axis (zero value) around the same year (1996), when the AMO switched from negative to positive phase while the PDO shifted from positive to negative phase. PC1 also changed around this time from generally negative to generally positive values. (Positive PC1 corresponds to decreasing sea-ice cover across the Arctic Ocean, and an overall increase in Antarctic sea ice when averaged across the Antarctic Ocean (Fig. 1a,b).) According to the Mann-Kendall test, the PC1, PDO and AMO time series all had a significant abrupt change in the late 1990s (not shown). Multiple linear regression analyses suggest that PC1 has a statistically significant relationship to PDO and AMO for all seasons. The regression coefficients (significant at 99% confidence level) (Table 2) are negative for PDO and positive to AMO regardless of the season. The regression coefficients with PDO are larger than the coefficients with AMO in DJF and MAM, suggesting a stronger response of PC1 to the changes in PDO in these months, and the opposite occurs in JJA and SON when PC1 responds more strongly to the changes of AMO than the changes in PDO.

The analyses presented above lend support for the proposed connectivity between the two polar regions of the trends in sea-ice concentration. The short length of the NSIDC sea-ice concentration time series is problematic, however. To further explore the proposed hypothesis, our second set of analyses focuses on long-term estimates of sea-ice concentration obtained from the Twentieth Century Reanalysis (V2) for the period 1856 through 2011^36^ and the Hadley Center sea-ice concentration estimates (HadISST) derived from multiple observation sources for the period 1870 to 2015^37^. Although of longer duration compared to the NSIDC time series, the HadISST dataset suffers, especially in the Antarctic, from the smaller number of observations in the pre-satellite era^37^. Also, the HadISST and Twentieth Century Reanalysis time series are not strictly independent as the HadISST sea-ice concentrations served as boundary conditions to the National Centers for Environmental Prediction (NCEP) atmosphere-land model used for the reanalysis^36^. Thus, the analyses below supplement rather than supersede the analyses for the short-term NSIDC record. The Twentieth Century Reanalysis and HadISST time series of sea-ice concentrations were initially detrended assuming a linear trend, and a 20-year low-pass filter was further applied. The AMO and PDO time series for 1856–2011 were similarly detrended and filtered.

The results of the analysis of the longer sea-ice concentration datasets also support a connection between the opposing trends in sea-ice concentrations in the Arctic and Antarctic and a relationship with the AMO and PDO. As for the NSIDC time series, EOF analysis was applied to the two time series. For brevity, only the results for SON, when the variance explained by EOF1 is largest, are discussed and displayed. For the Twentieth Century Reanalysis, EOF1 for SON (Fig. 4a) explains about 56% of the total variance of the global sea-ice cover and reveals opposite changes between the Arctic, dominated by negative anomalies, and the Antarctic, dominated by positive anomalies. The PC1 time series (Fig. 4b) shows a steadily decreasing trend from the 1860 to mid 1970s, with values switching from positive to negative in the mid-1980s. In the past four decades, PC1 rapidly increased at a rate more than four times the earlier decreasing rate. The correlations between the PC1 time series and the time series of the PDO and the AMO indices are −0.35 and 0.31 (both are significant at 99.9% confidence level), respectively. The multiple-linear regression of PC1 on PDO and AMO yields regression coefficients of −0.27 (for PDO) and 0.21 (for AMO), respectively (both are significant at the 99% confidence level). The signs of the regression coefficients are in the same direction as the signs obtained above for the NSIDC sea-ice concentrations.

The spatial patterns of EOF1 for the HadISST sea-ice concentration also display opposite anomalies for the two polar areas (negative for the Arctic and positive for the Antarctic) (Fig. 4a), although the spatial extent of the negative anomalies north of the Antarctic Peninsula is larger for HadISST than for either the Twentieth Century Reanalysis or the NSIDS observations. The PC1 time series (Fig. 4b) displays a sharp decreasing trend from the mid-1930s to 1950 in contrast to the gradual decrease seen for the Twentieth Century Reanalysis data, although the sharply increasing trend since approximately 1970 is evident for both datasets. The connection of the HadISST PC1 time series to PDO and AMO appears to be weaker as indicated by smaller correlation coefficients of −0.23 (98% confidence level) and 0.20 (99% confidence level) and regression coefficients only significant at 95% confidence level.

Lastly, we return to the NSIDC dataset to explore possible mechanisms for the global sea-ice trends. Sea-surface temperature (SST), 200-hPa geopotential height, and 2-m air temperature anomalies separately are regressed on PC1 obtained from the NSIDC time series of sea-ice concentrations. Here we only show the regression maps for SON (Fig. 5) since the results are similar for the other seasons. The SST regression map shows a spatial pattern that resembles the negative phase of PDO, with negative SST anomalies over the tropical central and eastern Pacific Ocean and the Southern Ocean, and positive SST anomalies over the tropical western Pacific Ocean and central Pacific Ocean (Fig. 5a). A SST pattern similar to the positive phase of AMO is seen in the northern Atlantic Ocean. Positive SST anomalies also occur over the Chukchi, East Siberian, and Beaufort Seas. Two wave trains, one in each hemisphere, are evident from the 200-Pa anomalies, likely excited by convective heating anomalies from the precipitation anomalies related to the anomalous SST over the tropical western Pacific Ocean^29^,^35^ (Fig. 5b). The Northern Hemisphere wave train exhibits positive 200 hPa geopotential height anomalies over several regions including the western North Pacific Ocean, eastern North America and the North Atlantic Ocean, eastern Europe, and the Arctic Ocean, and negative anomalies over the central and tropical Pacific Ocean and northwestern North America. The Southern Hemisphere wave train shows negative height anomalies over the Pacific portion of the Southern Ocean and the ocean south of Africa, and positive anomalies elsewhere over the Southern Hemisphere. Over the Arctic Ocean, the positive height anomalies may induce positive surface air temperature anomalies (Fig. 5c) through cloud-radiation feedback^14^, reducing sea-ice cover that, through ice-albedo feeback^13^, further enhances the warming. Similar feedback mechanisms likely also play an important role in the Antarctic. The positive height anomalies generate warm surface-temperature anomalies resulting in the sea-ice decline, and vice versa. In addition, the sea-ice decline on the Bellingshausen and Weddell Seas is associated with the anomalous northerly winds there (not shown). Similar relationships between changes in sea-ice cover and the PDO and the AMO exist for the other seasons.

We hypothesized that sea ice loss in the Arctic and net sea ice gain in the Antarctic during recent decades are connected via global climate oscillations, namely the AMO and PDO, and provided an initial assessment of the proposed hypothesis. Although this assessment is constrained by the limitations of the observational record of sea-ice concentrations, the congruent findings from statistical analysis of three time series of sea-ice concentration of varying length and resolution (i.e., NSIDC, HadISST, and Twentieth Century Reanalysis) point to a possible teleconnection between the opposite changing trends in sea-ice coverage in the two polar regions. These findings are consistent with those from previous studies that focused separately on either the north or the south polar regions. For example, several studies suggested that the changes in the SST over the tropical Pacific^23^,^24^,^25^,^26^,^27^,^28^,^29^ and Atlantic Oceans^31^,^32^,^33^,^34^ related to the AMO and PDO contribute to the increased Antarctic sea ice in the last three decades, and other studies have linked the decreasing trends of Arctic sea ice to PDO and AMO^16^,^19^,^20^. These results, together with our findings that simultaneously consider sea-ice coverage in both polar regions, help improve the understanding of the role of interdecadal climate modes (AMO and PDO) on trends in oceanic and atmospheric variables over the polar regions. Our analyses are based solely on statistical considerations, and further analysis, including numerical modeling, is necessary to fully understand this potential teleconnection and other underlying physical mechanisms for the opposing trends in the Arctic and Antarctic sea-ice concentrations.

## Methods

Three data sets of sea-ice concentration were used in this study. The first dataset was obtained from the National Snow and Ice Data Center (NSIDC) in Boulder, Colorado (http://nsidc.org/data/NSIDC-0051). The sea-ice concentrations are derived from the Nimbus-7 Scanning Multichannel Microwave Radiometer (SMMR), the Special Sensor Microwave/Imagers (SSM/I) on the F8, F11, and F13 satellites, and the Special Sensor Microwave Imager/Sounder (SSMI/S) on the F17 satellite of the Defense Meteorological Satellite Program (DMSP). The NSIDC dataset, available daily from 26 October 1978 to the present, has a grid cell resolution of 25 × 25 km in a polar stereographic projection. Sea-ice concentration, defined as the areal percentage covered with ice, is generated using the National Aeronautics and Space Administration (NASA) team algorithm developed by the Oceans and Ice Branch, Laboratory for Hydrospheric Processes at NASA Goddard Space Flight Center (GSFC). More details are available from the NSIDC web site. Although the NSIDC dataset has been utilized widely to understand the variability of sea ice in Arctic and Antarctic, it has some limitations that may affect the interpretation of the results from the current study. First, and most important to our analysis, the NSIDC dataset is of relatively short length, especially in comparison to the low-frequency modes of climate variability. Second, snow and surface flooding on sea ice and seasonal variations in the physical characteristics of sea ice may influence the accuracy of the sea-ice data^38^. Third, land contamination and weather effects may decrease the accuracy of the sea-ice data^39^.

The NSIDC observed sea-ice data were supplemented by long-term sea-ice data from the Hadley Centre Sea Ice and Sea Surface Temperature (HadISST) data set^37^ and the Twentieth Century Reanalysis (V2)^36^. The HadISST provides monthly globally-complete fields of sea-ice concentration from 1870 –present on a 1° × 1° degree latitude-longitude grid. This data set combines multiple sources of sea-ice fields, including historical sea charts from shipping and expeditions, satellite retrievals, and operational analyses. A primary concern of the HadISST dataset is heterogeneities introduced into the time series by the quality of the different sources of sea-ice information and their varying spatial coverage and density. The coarse spatial and temporal resolution is also a limitation. The Twentieth Century Reanalysis was generated by assimilating surface pressure and sea-level pressure observations into an Ensemble Kalman Filter data assimilation system and using the National Center for Environmental Prediction (NCEP) atmosphere-land model to generate first-guess fields with the HadISST sea ice and SST fields as prescribed boundary conditions^36^. This dataset has a fine (6-hourly) temporal resolution but a coarse (2° latitude-longitude) spatial resolution.

The large-scale atmospheric circulations were derived using data from the National Centers for Environmental Prediction (NCEP)-Department of Energy (DOE) global reanalysis^40^. The National Oceanic and Atmospheric Administration (NOAA) Extended Reconstructed Sea Surface Temperature (SST) dataset^41^ was used for identifying the SST patterns over the global ocean. The monthly PDO indices^42^ were obtained from University of Washington (http://www.atmos.washington.edu/~mantua/abst.PDO.html) and the monthly AMO indices^43^ were downloaded from NOAA (http://www.esrl.noaa.gov/psd/data/correlation/amon.us.data).

The empirical orthogonal function (EOF) technique was used to reveal the dominant patterns of the variability of the seasonal mean sea-ice concentrations in the two polar regions. The EOF analysis can produce a set of modes that consist of spatial patterns (EOFs) and corresponding time series [principal components (PCs)]. For each mode, its EOF and PC are orthogonal to the EOFs and PCs of all other modes. Each mode has a corresponding eigenvalue that depicts the percentage of variance explained by the mode. In this study the EOF technique was utilized to identify the prevailing spatial patterns and time variations of seasonal-mean sea-ice concentration for MAM (March-May), JJA (June-August JJA), SON (September-November SON), and DJF (December-February DJF). Finally, regression analysis was used to explain the large-scale circulation anomalies associated with the leading EOF patterns. Specifically, a large-scale circulation variable (e.g., 200-hPa geopotential height or SST) was regressed to the leading PC time series to explore the origin of the leading EOF patterns. This latter analysis was limited to only the higher resolution NSIDC sea-ice concentration data set.

## Additional Information

**How to cite this article:** Yu, L. *et al*. Possible connections of the opposite trends in Arctic and Antarctic sea-ice cover. *Sci. Rep.* **7**, 45804; doi: 10.1038/srep45804 (2017).

**Publisher's note:** Springer Nature remains neutral with regard to jurisdictional claims in published maps and institutional affiliations.

## Figures and Tables

**Figure 1:**
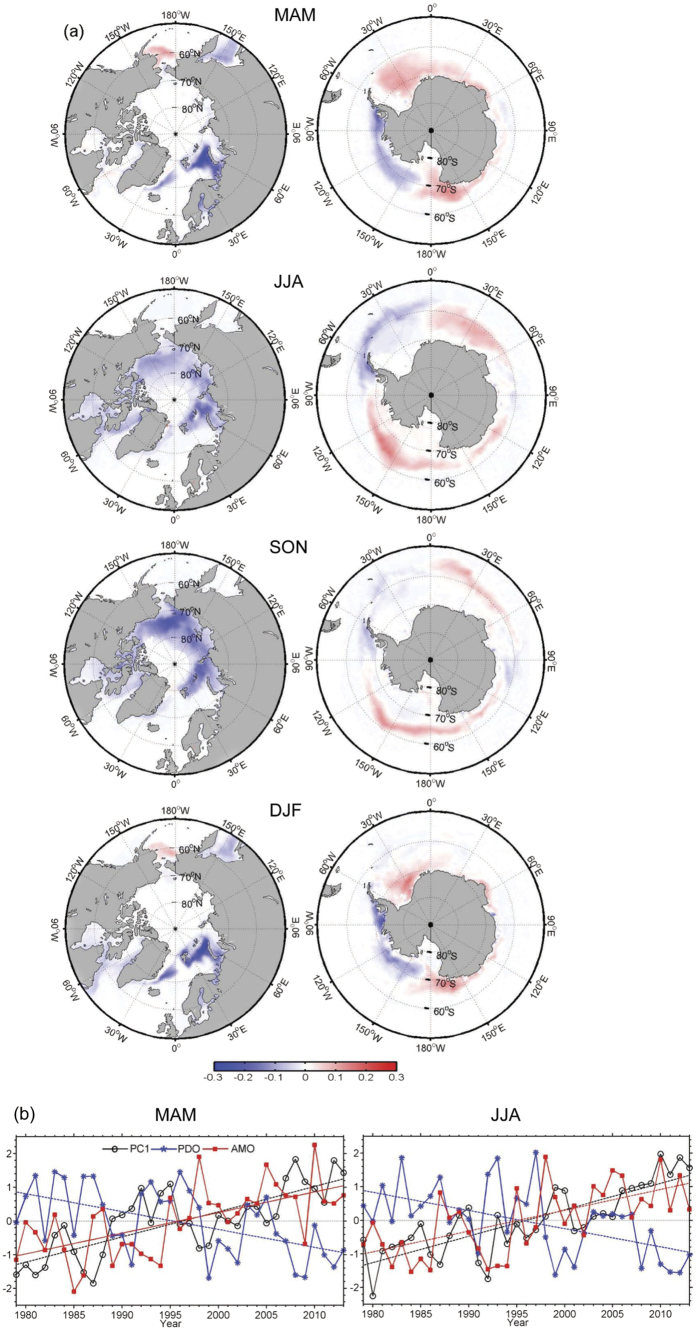
Spatial patterns (EOFs) (**a**) and time coefficients (PCs) (**b**) of the first EOF modes of global sea ice concentration for the regions of 50°–90°N (left) and 50°–90°S (right) for the 1979–2013 period for MAM (March, April, May), JJA (June, July, August), SON (September, October, November) and DJF (December, January, February). Also shown in (**b**) are the time series of the PDO (blue) and AMO (red) indices. The dashed and dotted lines in (**b**) refer to the trends and the zero lines. This figure is created using MATLAB & Simulink Release 2010b (www. mathworks.com).

**Figure 2:**
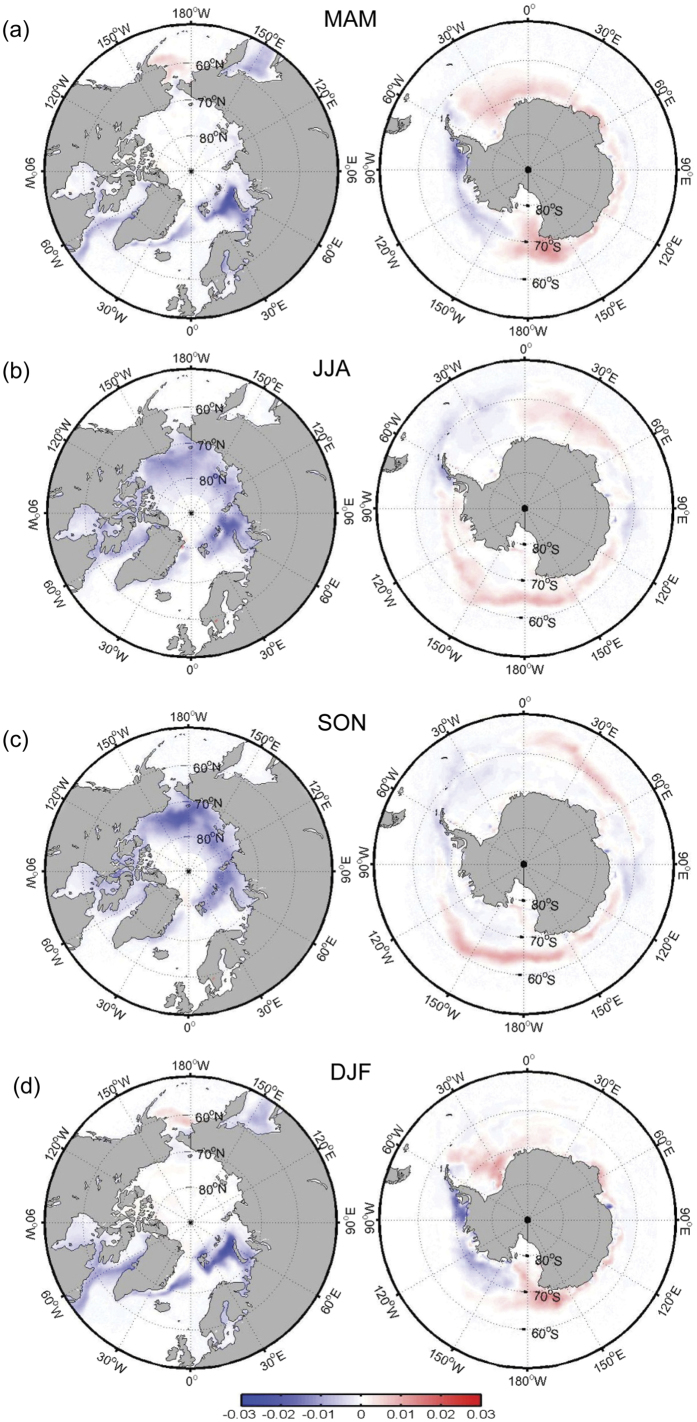
Trends (per year) in sea ice concentration for the regions of 50°–90°N (left) and 50°–90°S (right) for the 1979–2013 period for MAM (**a**), JJA (**b**), SON (**c**) and DJF (**d**). This figure is created using MATLAB & Simulink Release 2010b (www. mathworks.com).

**Figure 3:**
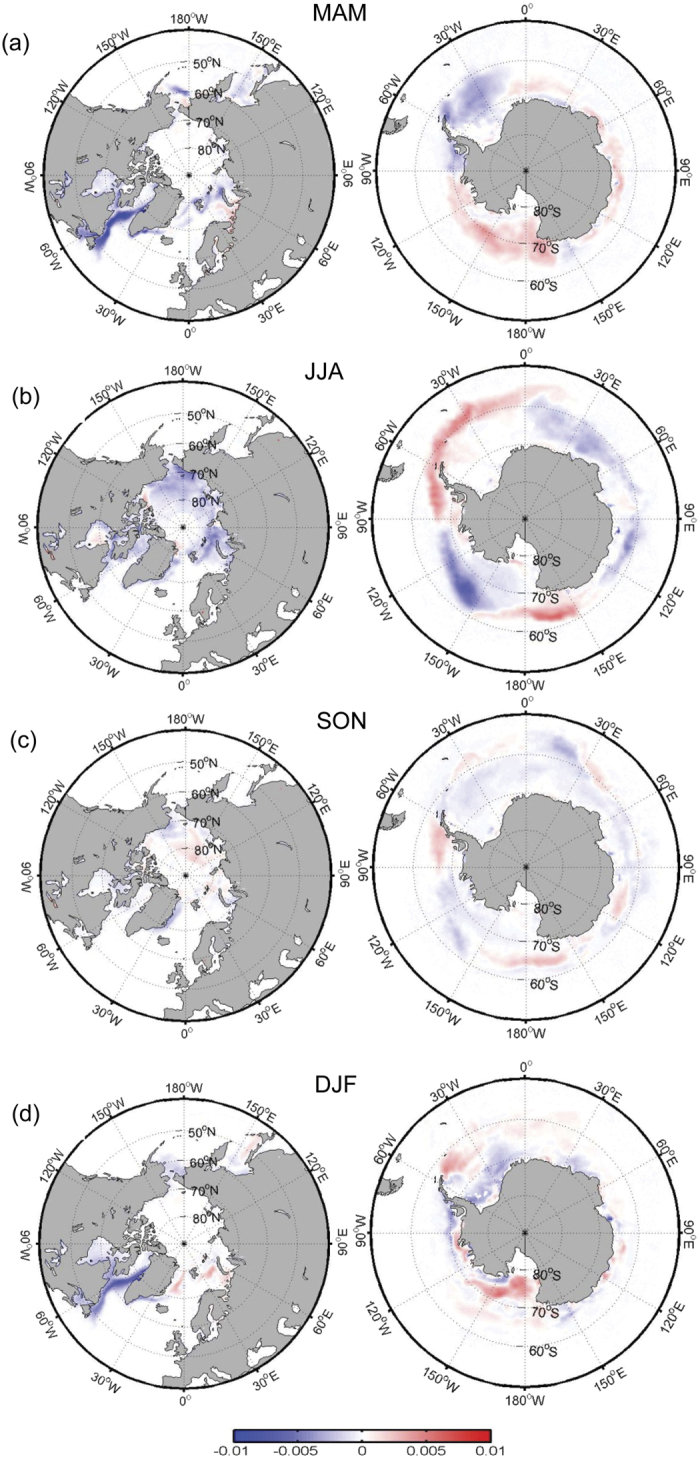
Residual trends in sea ice concentration for 50°–90°N (left) and 50°–90°S (right) for the 1979–2013 period for MAM (**a**), JJA (**b**), SON (**c**) and DJF (**d**). This figure is created using MATLAB & Simulink Release 2010b (www. mathworks.com).

**Figure 4:**
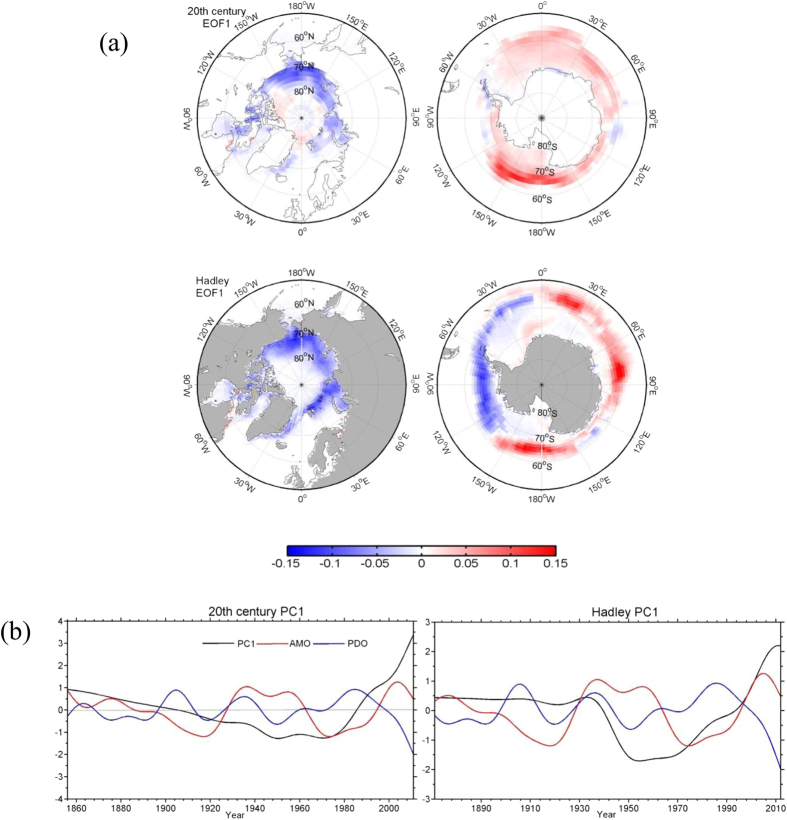
Spatial patterns (EOFs) (**a**) and time coefficients (PCs) (**b**) of the first EOF modes of global sea ice concentration (50°–90°N (left) and 50°–90°S (right)) from the Twentieth Century Reanalysis (V2) (1856–2011) and the Hadley Center (1870–2015) data in autumn (September, October and November). The black, blue and red lines refer to PC1s, the PDO indices and the AMO indices, respectively. This figure is created using MATLAB & Simulink Release 2010b (www. mathworks.com).

**Figure 5:**
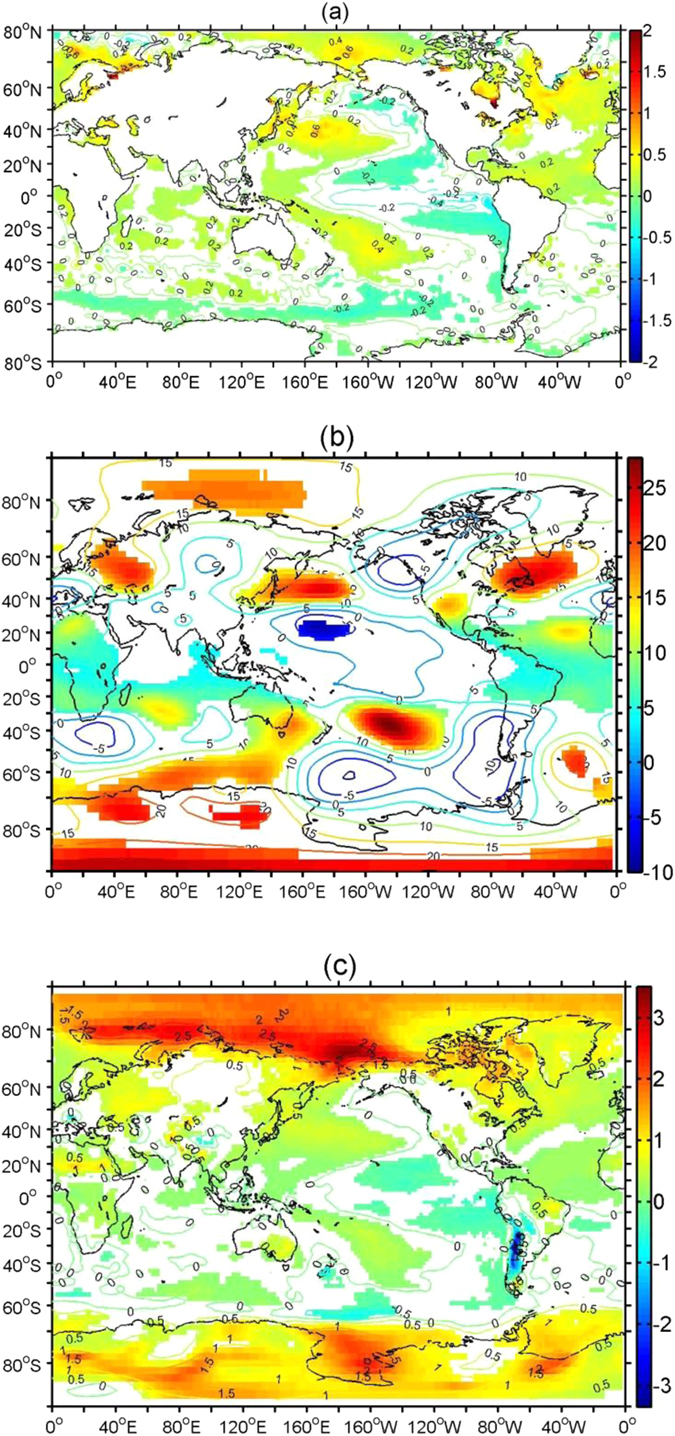
The anomalous SST (°C) (**a**), 200 hPa geopotential height (gpm) (**b**) and 2-m air temperature (°C) (**c**) regressed to the time series of the first EOF mode of sea ice concentration for SON for the 1979–2013 period. The filled regions are significant above the 95% confidence level. This figure is created using MATLAB & Simulink Release 2010b (www. mathworks.com).

**Table 1:** Trends in the PC1 for the sea ice concentration from the NSDIS data, the PDO indices and the AMO indices for the period 1979 to 2013 for each season with greater than 99.9% confidence level.

	MAM	JJA	SON	DJF
PC1	0.0749	0.0789	0.0861	0.0875
PDO	−0.0530	−0.0542	−0.0651	−0.0594
AMO	0.0614	0.0621	0.0739	0.0695

**Table 2:** Linear regression coefficients of PC1 for se ice concentration from NSDIS data for the period 1979 to 2013 on PDO and AMO. *PC*1 = *K*0 + *K*1 × *PD* + *K*2 × *AMO* All regressions were significant at the 99% confidence interval. K1 and K2 for each season are significant at the 99% confidence level.

	K1	K2
MAM	−0.4167	0.2465
JJA	−0.3897	0.4035
SON	−0.4009	0.4608
DJF	−0.5663	0.3230
